# Assessment of heat stress in 7-week old dairy calves with non-invasive physiological parameters in different thermal environments

**DOI:** 10.1371/journal.pone.0200622

**Published:** 2018-07-18

**Authors:** Levente Kovács, Fruzsina Luca Kézér, Ferenc Ruff, Viktor Jurkovich, Ottó Szenci

**Affiliations:** 1 MTA–SZIE Large Animal Clinical Research Group, Üllő-Dóra major, Hungary; 2 Institute of Animal Husbandry, Faculty of Agricultural and Environmental Science, Szent István University, Gödöllő, Hungary; 3 Department of Methodology, Hungarian Central Statistical Office, Budapest, Hungary; 4 Department of Animal Hygiene, Herd Health and Veterinary Ethology, University of Veterinary Medicine, Budapest, Hungary; University of Illinois, UNITED STATES

## Abstract

We estimated thermal stress in 7-week old Holstein bull calves during a warm episode in summer to study acute physiological responses of calves to heat stress. Data were collected over a 5-day period: day 1 (control), day 2 (heat stress), and a 3-day post-stress period in shaded (n = 8) and unshaded (n = 8) thermal environments. On the control day, both groups were shaded. Thermal environment was characterized by relative humidity, ambient temperature, and the temperature–humidity index (THI). Physiological variables included respiratory rate, rectal temperature, ear skin temperature and heart rate. Correlations between animal-based and meteorological indices were calculated, and ambient temperature correlated slightly better with physiological measures than THI. Rectal temperature was the only animal-based parameter that showed stronger correlations with the thermal indices when calculated for the shaded than for the unshaded environment [r = 0.42 vs. r = 0.47, *P* = 0.032 (ambient temperature), r = –0.39 vs. r = –0.45, *P* = 0.012 *P* = 0.015 (relative humidity), r = 0.41 vs. r = 0.46, *P* = 0.022 (THI)]. No differences were found between groups during the control day for any of the physiological parameters. During days 2 and 3, average and maximal values of respiratory and heart rates were higher in unshaded calves than in shaded ones. Maximal respiratory rates were in average by 25.9, 17.8 and 10.1 breaths/min lower in shaded calves than in unshaded calves for days 2, 3 and 4, respectively (*P* < 0.001, *P* < 0.001 and *P* = 0.024). Maximal heart rate was 127.4 ± 8.5 vs. 99.2 ± 6.3 beats/min on the heat stress day (*P* < 0.001), and 121.0 ± 6.9 vs. 103.4 ± 7.7 beats/min on day 3 (*P* = 0.006) in unshaded and shaded calves, respectively. Maximal body temperatures were higher measured either in the rectum or on the ear skin in unshaded calves than in shaded ones (with 0.5 and 1.6°C, *P* = 0.040 and *P* = 0.018, respectively), but only on the heat stress day. Based on our results, shading of young calves may be adequate for alleviating acute heat stress in continental regions. Ambient temperature is appropriate to estimate acute heat stress in dairy calves.

## Introduction

A persistent rise in global mean temperature has severe impacts on natural and societal systems [1]. It has been shown that the increased tendency of heat-related mortality causes a considerable economic loss for the dairy industry in regions where high environmental temperatures represent a concern [2, 3]. Although animal-friendly housing technologies have been introduced to alleviate weather-related heat exposure in dairy calves [4], young animals are commonly kept in individual polyethylene hutches with a limited outdoor space. Although these pens allow calves freedom to select a comfortable resting place other than in the hutch, animals are exposed to direct sunlight and are at risk during warm episodes of summer.

Based on ambient temperature and relative humidity, thermos-hygrometric indices are used for the assessment of the thermal environment in cattle. The temperature-humidity index (THI) represents the combined effects of air temperature and relative humidity to estimate the level of thermal stress in adult cows [5].

In dairy [4, 6] and beef calves [7], the effects of thermal stress can be assessed using rectal temperature and respiratory rate. It is not known if there are physiological measures other than rectal temperature or respiratory rate indicative for acute heat stress. In this study, we monitored a typical warm episode that occurs more and more frequently in Central Europe during summer to assess acute physiological responses to heat stress of dairy calves in shaded and unshaded environments. We measured rectal temperature, respiratory rate, heart rate and ear skin temperature to assess the associations between physiological indices, ambient temperature, relative humidity and THI. We hypothesized that respiratory rate, ear skin temperature and heart rate will show higher correlations with meteorological parameters than rectal temperature, and correlations between animal-based and meteorological measures will differ based on the thermal environment (shaded vs. unshaded). We also hypothesized lower acute stress responses of calves kept in shaded hutches compared to non-shaded ones.

## Materials and methods

All sampling procedures and experimental manipulations with animals were specifically approved by the Pest County Government Office, Hungary, Department of Animal Health (Permit Number: PE/EA/1973-6/2016).

### Animals and experimental design

The experiment was carried out at a large-scale dairy farm in Hungary (N47°18'191'' E18°48'336''), which has a herd of 1000 lactating Holstein cows. The farm was visited between August 15 and 20 in 2016. As a field study, our experiment was timed based on the expected meteorological patterns of the regional weather forecast. The 6-day study started with a 24-h habituation to the study environment when preparation of the shading structure over the hutches of both shaded (n = 8) and unshaded calves (n = 8) were done. On day 1 (control, maximum of 28.3°C for the hutch environment) all calves were shaded for 24-h from 0:00 o’clock until 24:00 o’clock and shade was removed from the unshaded group at 24:00 o’clock. Day 2 was considered as the heat stress day (maximum of 37.7°C for the shaded hutch environment), and days 3–5 covered a post-stress period (day 3: maximum of 30.3°C, day 4: maximum of 26.5°C, and day 5 maximum of 24.3°C for the shaded hutch environment).

On the study farm, Holstein calves were housed individually in 1.65 × 1.20 m plastic calf hutches (Calf-Tel ECO, Hampel Animal Care, WI, USA) with a 1.60-m^2^ exercise pen that were arranged in rows based on their sex (heifer row and bull row) and birth date. To eliminate the possible effect of individual-related (i.e. sex, age and body weight) and environmental factors (i.e. row orientation) on our results, bull calves with similar age and body weight (means ± SD; age = 46.7 ± 2.4 days, body weight = 74.3 ± 2.6 kg) one week before weaning from the same row were selected for the study and assigned to the shaded or unshaded groups. The shading structure measured 32.5 × 3.4 m and covered the experimental hutches and pens as well. After removing the shading structure above unshaded calves, their hutches were exposed to direct sunlight 5 m from the shaded environment. A green raschel net (Nortene Texanet, Celloplast S.A.S, Ballée, France), located 1.9 m above the ground was used as shading material that provided 80% shading, according to the manufacturer. Calves received one feeding of 3.8 liters of milk replacer in the morning (5:00 o’clock) and had ad libitum access to chopped alfalfa hay and the starter grain diet (Purina calf starter, Cargill, USA), which met the requirements for preweaned Holstein calves [8]. Water from a plastic bucket (7.6 liters), filled twice a day, was provided throughout the study. The diet did not change throughout the experiment.

### Meteorological data

Ambient temperature and relative humidity were assessed for each calf by using the VOLTCRAFT DL-181THP device (Conrad Electronic SE, Hirschau, Germany) placed in the back of the hutches and with the Testo 175 H1 (Testo Inc., Sparta, USA) device that were fitted onto the shading structure 1 m above the ground above the exercise pens. A 30-min recording frequency was chosen (S1 Table) for both devices between day 1 0:00 o’clock (first recording) and day 5 24:00 o’clock (last recording). The position of the calves was retrospectively determined based on video recordings of two day/night outdoor network bullet cameras (Vivotek IP8331, VIVOTEK Inc., Taiwan) installed above the experimental area to allow the selection of the appropriate temperature and humidity values (recorded in the hutch or in the exercise pen) for the calculation of meteorological metrics. The thermal environment of the calves was characterized by temperature, relative humidity and THI. The THI was calculated by the formula described by Bianca et al. [9]:

THI=(0.35×Tdb+0.65×Twb)×1.8+32

where T_db_ = dry bulb temperature and T_wb_ = wet bulb temperature.

### Physiological data

Respiratory rate (breaths/min) was recorded by counting the movements of the abdominal muscles in the flanks during respiration [4], while calves were in a lying posture between day 1 0:00 o’clock (first observation) and day 5 24:00 o’clock (last observation) with a 4-h sampling frequency. Immediately after respiratory rate observation, rectal temperature was measured with a 10-sec digital thermometer (Digi-Vet SC 12; Jørgen Kruuse A/S, Langeskov, Denmark). Ear skin temperature was measured parallel with rectal temperature using the Testo 830 T2 infrared thermometer (Testo Inc., Sparta, USA).

Heart rate was recorded continuously between day 1 0:00 o’clock and day 5 24:00 o’clock using a Polar electrode belt with two integrated electrodes, a compatible Polar H7 HR sensor and a Polar V800 HR receiver (POLAR, Kempele, Finland). Devices were fitted to animals on day 0 and were removed from the calves after day 5 then heart rate data were transmitted to the Polar FlowSych program. The analysis of heart rate was performed with the Kubios HRV standard software (version 2.2, Biomedical Signal Analysis Group, Department of Applied Physics, University of Kuopio, Finland) using equal lengths of 5-min samples recorded for undisturbed lying posture of the animals. Posture was recorded using the HOBO Pendant G data logger (Onset Computer Corporation, Bourne, MA), which was validated for dairy calves [10].

### Statistical evaluation

All statistical analyses were performed in the R–3.0.2. statistical environment and language [11]. Data were tested for constant variance (Levene’s test) and the Shapiro–Wilk test was used for testing the equality of error variances both for shaded and unshaded groups.

For the evaluation of the associations between animal-based heat stress indicators and thermal parameters, data recorded from both shaded and unshaded calves were used. Thermal indices were calculated from meteorological data recorded by devices fitted in the hutch or in the pen based on the location of the calves. Pearson correlations were calculated based on individual observations between day 1 0:00 o’clock and day 5 24:00 o’clock [for heart rate with 30-min sampling frequency (n = 3840); for respiratory rate, rectal and ear skin temperature with 4-h sampling frequency (n = 480)]. We tested whether a difference between correlation coefficients exists according to the environment (shaded or unshaded). For this purpose, the Fisher-type *z*-transformation based *z*-test was used at the significance level of 0.05.

To evaluate the effect of shading on the thermal environment of the animals, maximal and average values of selected thermal indices were calculated for all experimental days and compared between unshaded and shaded groups in the hutch environment, using all recorded meteorological data with 30-min sampling frequency. Statistical comparisons between environments were made with the *t*-test at the significance level of 0.05.

The effect of shading was tested by comparing physiological responses of shaded and unshaded groups by using the individuals’ maximum and average values of respiratory rate, heart rate, ear skin and rectal temperatures calculated for each experimental day. All recorded animal-based data were used in this analysis, irrespective of the location of the calves (measured either in the hutch or the pen). Statistical comparisons between groups were made with the *t*-test. Significance was set at the level of 0.05.

## Results and discussion

### Relationships between thermal and physiological measures

**Fig 1:**
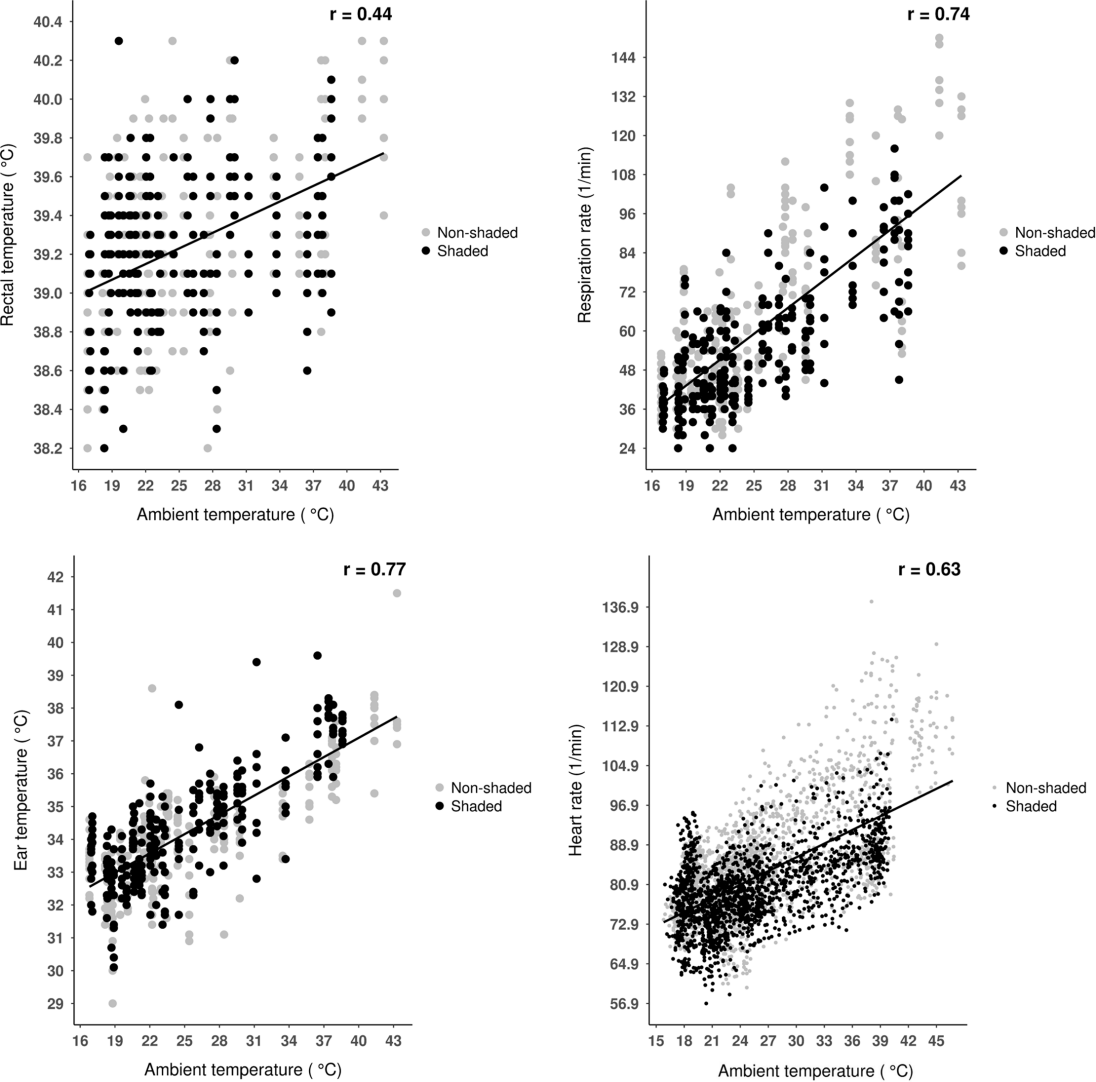
Relationship between rectal temperature, respiratory rate, ear temperature, heart rate and ambient temperature in dairy calves. Points represent individual observations (n = 480 for rectal temperature, ear temperature and respiratory rate; n = 3840 for heart rate) between day 1 0:00 o’clock and day 5 24:00 0’clock; lines represent simple linear regression equations, and ‘r’ represents the correlation coefficient.

**Fig 2:**
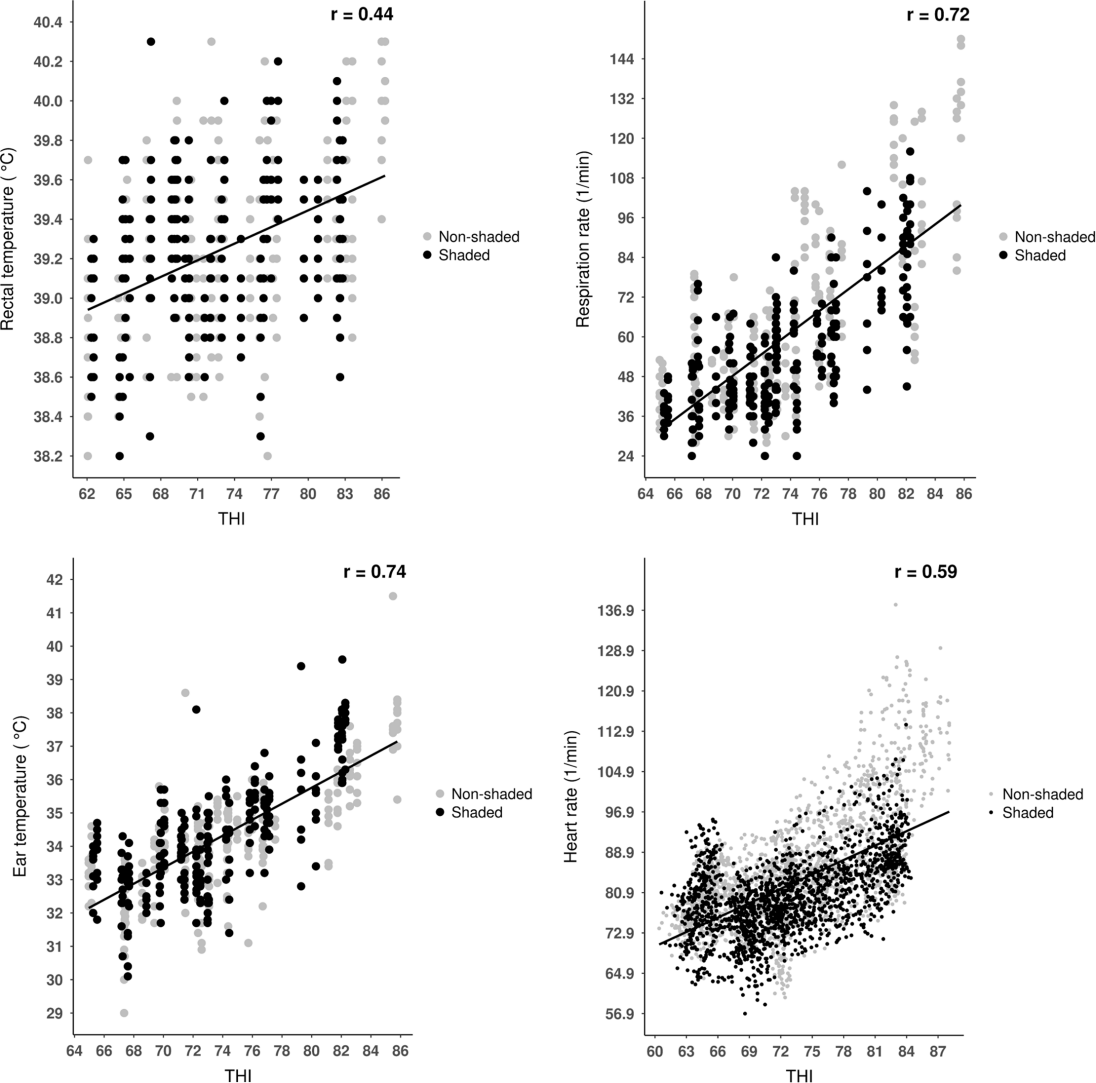
Relationship between rectal temperature, respiratory rate, ear temperature, heart rate and relative humidity in dairy calves. Points represent individual observations (n = 480 for rectal temperature, ear temperature and respiratory rate; n = 3840 for heart rate) between day 1 0:00 o’clock and day 5 24:00 0’clock; lines represent simple linear regression equations, and ‘r’ represents the correlation coefficient.

Generally, animal-based heat stress indicators showed strong positive correlation with ambient temperature and THI (**Figs 1** and **2**). The THI formula used in our study was found to be appropriate for continental weather characteristics (low daytime relative humidity) in dairy cows [12,13], and in this formula relative humidity (wet bulb temperature) is weighted to a lower degree. This could be the explanation of the similar (or slightly higher in cases of ear skin temperature and heart rate) correlations of ambient temperature with physiological indices as THI in our study. Based on these findings, the use of simple temperature can be suggested for the estimation of acute heat stress experienced by dairy calves.

**Fig 3:**
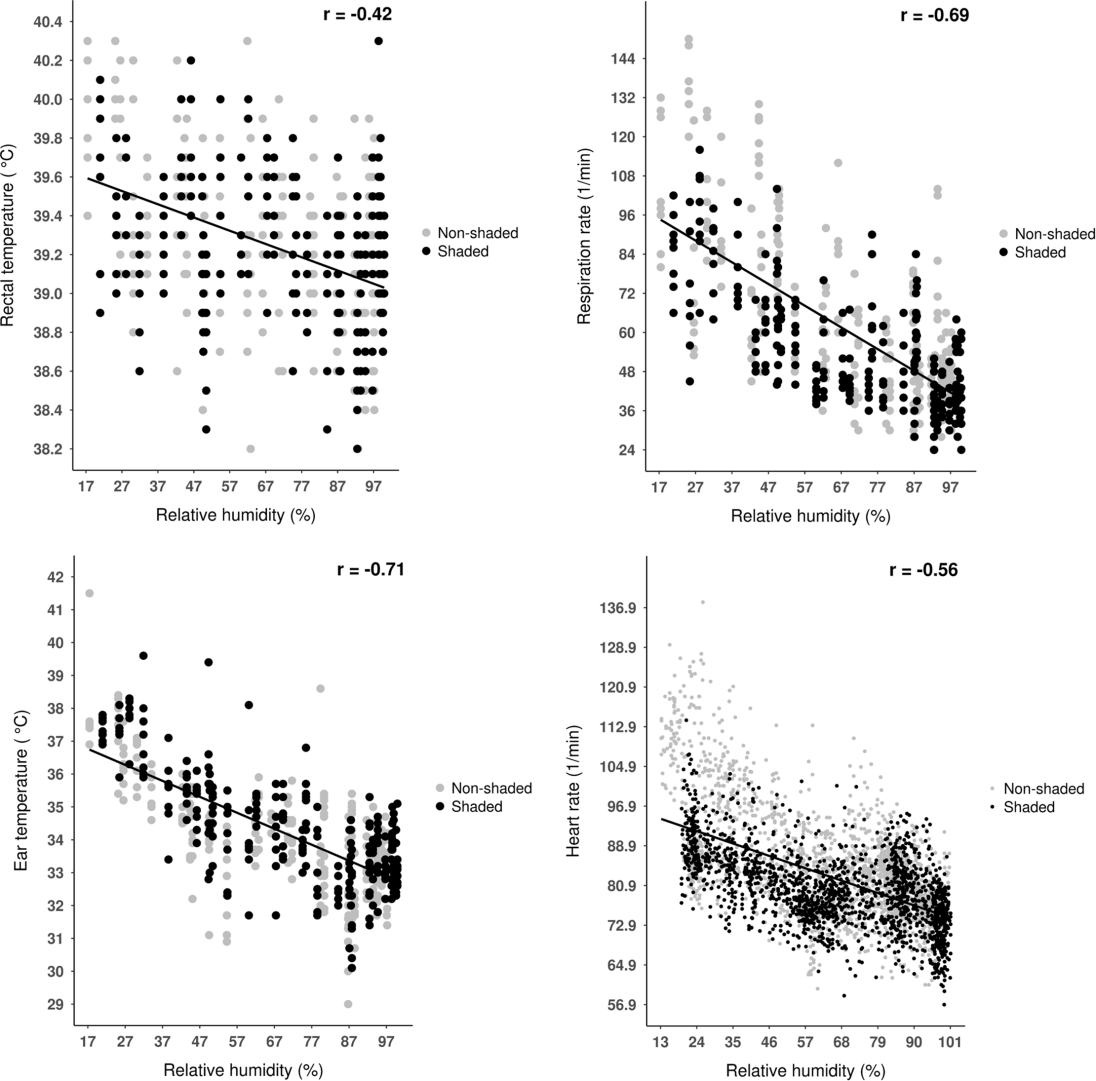
Relationship between rectal temperature, respiratory rate, ear temperature, heart rate and THI in dairy calves. Points represent individual observations (n = 480 for rectal temperature, ear temperature and respiratory rate; n = 3840 for heart rate) between day 1 0:00 o’clock and day 5 24:00 0’clock; lines represent simple linear regression equations, and ‘r’ represents the correlation coefficient.

**Fig 4:**
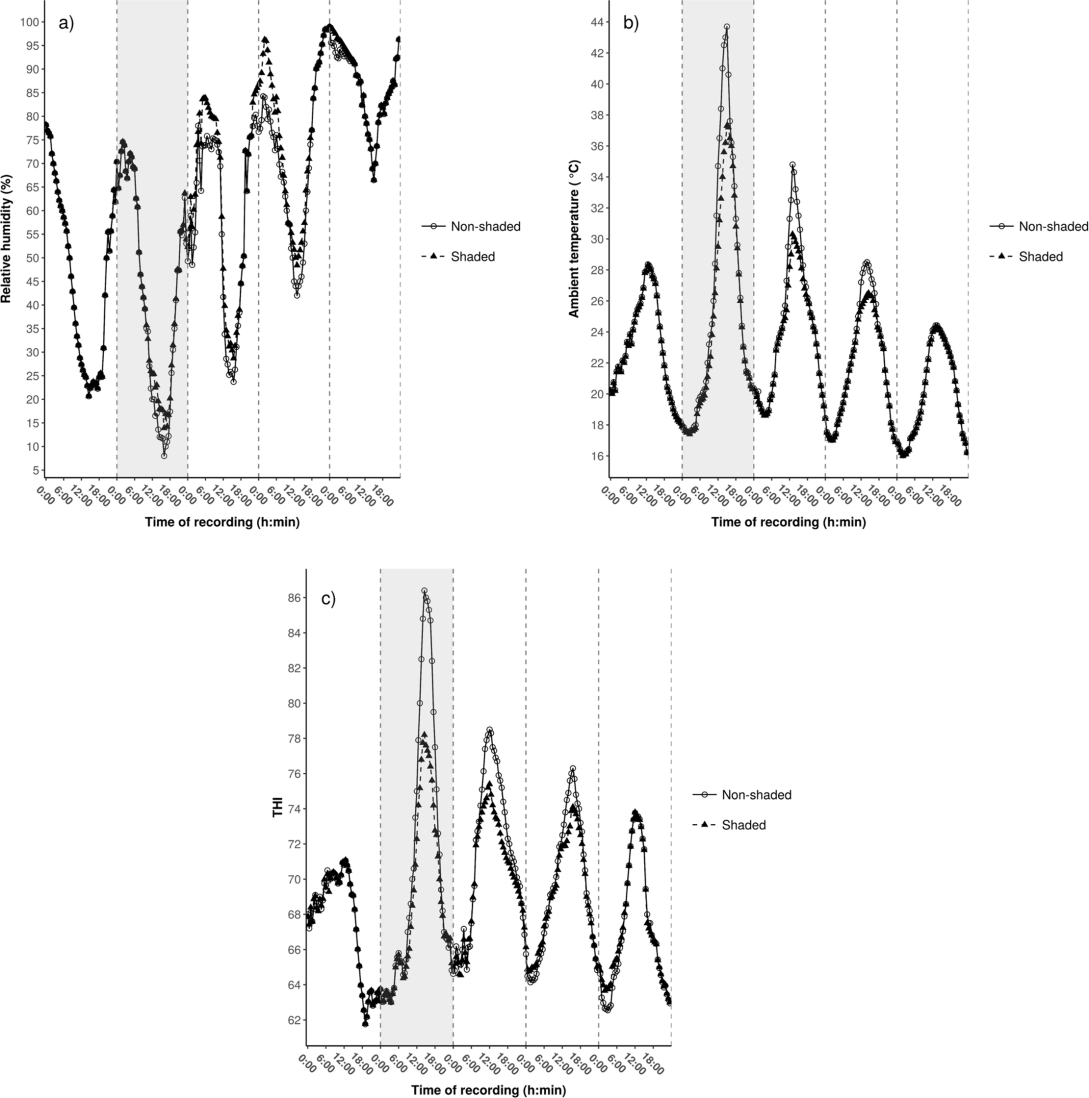
**Changes in relative humidity (a), ambient temperature (b) and THI (c) in the shaded and unshaded hutch environment during the 5-day study period.** Data are presented with a 30-min recording interval. The grey area between the first and the second dashed vertical lines represents day 2 (heat stress day).

Animal-based measures showed a negative correlation with relative humidity (**Fig 3**). During the experimental period, relative humidity and ambient temperature showed a converse pattern with increasing relative humidity and decreasing ambient temperature during the nighttime and increasing ambient temperature during the daytime (see **Fig 4A** and **4B** later). Thus, the negative relationship between relative humidity and animal-based indices assuredly mirrored the diurnal changes of ambient temperature as well not the direct effect of relative humidity itself. Dikmen and Hansen [5] found also negative correlation (r = –0.38) between rectal temperature and relative humidity in dairy cows. Effects of relative humidity on physiological measures should be estimated using climate-controlled chambers, where constant temperature and fluctuating humidity values could be set up.

The only correlation study that investigated temperature indices with respect to hyperthermia used only rectal temperature as an animal-based variable in adult cows [5]. A small number of studies on calves that measured rectal temperatures or respiratory rates used THI as environmental variable. It is also important to note, that data for the current study were collected in a continental region, in which low daytime humidity is a prevailing characteristic, while the referred experiments were done in tropical [14,15] or humid subtropical environments [4], therefore, comparison of the present results with earlier data is difficult.

It was hypothesized that rectal temperature would less correlate with meteorological parameters than other physiological measures. Our hypotheses were supported, since when data of both shaded and unshaded environments were assessed, rectal temperature showed medium strong, while other physiological parameters showed strong correlations with meteorological measures (**Figs 1–3**). This suggests that respiratory rate, heart rate or ear skin temperature could be more appropriate to estimate acute heat stress than rectal temperature in dairy calves. Our second hypothesis in that thermal environment would affect correlations between physiological and meteorological measures was partly supported. Interestingly, rectal temperature was the only animal-based parameter that showed stronger correlations with the thermal indices when calculated for the unshaded than for the shaded environment [r = 0.42 vs. r = 0.47, *P* = 0.032 (ambient temperature), r = –0.39 vs. r = –0.45, *P* = 0.012 *P* = 0.015 (relative humidity), r = 0.41 vs. r = 0.46, *P* = 0.022 (THI)]. Any other correlations showed no significant differences between shaded and unshaded environments (*P* > 0.05 for all cases). An earlier study reported an r = 0.52 correlation between THI and rectal temperature in dairy cows both recorded between 15:00 and 17:00 o’clock, under high ambient temperatures [5]. In the light of the authors’ results, our findings point out that the associations between rectal temperatures and environmental heat stress indices could be stronger with higher heat load, which should be considered in future studies on calf thermal comfort.

### Effect of shading on the thermal environment

Changes in relative humidity, ambient temperature and THI in the shaded and unshaded hutch environments over the study period are presented in **Fig 4A–4C**.

There were no statistical differences either for maximal or average values of relative humidity between shaded and unshaded environments during the experimental period. On day 1 (control) no differences were found in maximal and daily average values either for ambient temperature (*P* = 0.875 and *P* = 0.920) or THI (*P* = 0.930 and *P* = 0.945). Measurements between day 2 0:00 o’clock and day 4 24:00 o’clock proved the positive effect of shading on the thermal environment. On day 2, maximal temperature and THI were observed at 16:00 o’clock in the unshaded hutch environment that were higher than those recorded for the shaded one [43.7 ± 0.1 vs. 37.7 ± 0.1°C; *P* = 0.005 (**Fig 4B**) and 86.4 ± 0.1 vs. 78.2 ± 0.1; *P* < 0.001 (**Fig 4C**), respectively]. Reduced heat load in the shaded hutch environment compared to the unshaded one was reflected by daily averages of temperature as well on the heat stress day (28.5 ± 0.1 vs. 24.2 ± 0.1°C; *P* < 0.001) and THI (78.1 ± 0.1 vs. 71.3 ± 0.1; *P* = 0.011). Earlier studies have shown that shading reduces hutch temperature by around 1 to 2°C [6, 13]; however, in these experiments temperature was measured only at 15:00 o’clock. Maximums and daily averages of ambient temperature were lower in the shaded environment compared to the unshaded one on days 3 (*P* < 0.001 and *P* = 0.005) and 4 (*P* = 0.008 and *P* = 0.012), respectively. Maximal and average THI showed similar differences between the shaded and unshaded environments for the first two days of the post-stress period (day 3: *P* = 0.003 and *P* = 0.014; day 4: *P* = 0.015 and *P* = 0.026). On day 5 no differences were found in maximal and daily average values either for ambient temperature (*P* = 0.395 and *P* = 0.420) or THI (*P* = 0.526 and *P* = 0.545).

### Effect of shading on physiological parameters

Recent studies on heat stress of dairy calves tested different housing methods [4, 16] or compared the stress-reducing efficiency of artificial vs. natural shade [14]; all using only rectal temperature and respiratory rate as indicators of heat stress. Here, the analysis was focused on ear temperature and heart rate as well that could be measured non-invasively and inexpensively both having the potential to assess heat stress in non-producing young calves. We were particularly interested in acute heat stress responses of calves during a typical warm episode in summer. Physiological reactions recorded over a longer period might influenced reactions by adaptation, development of calves during this early age of life, changes in feed (i.e. removal of milk during weaning) or meteorological effects such as a prolonged decline in temperature or cold and hot fronts.

**Fig 5:**
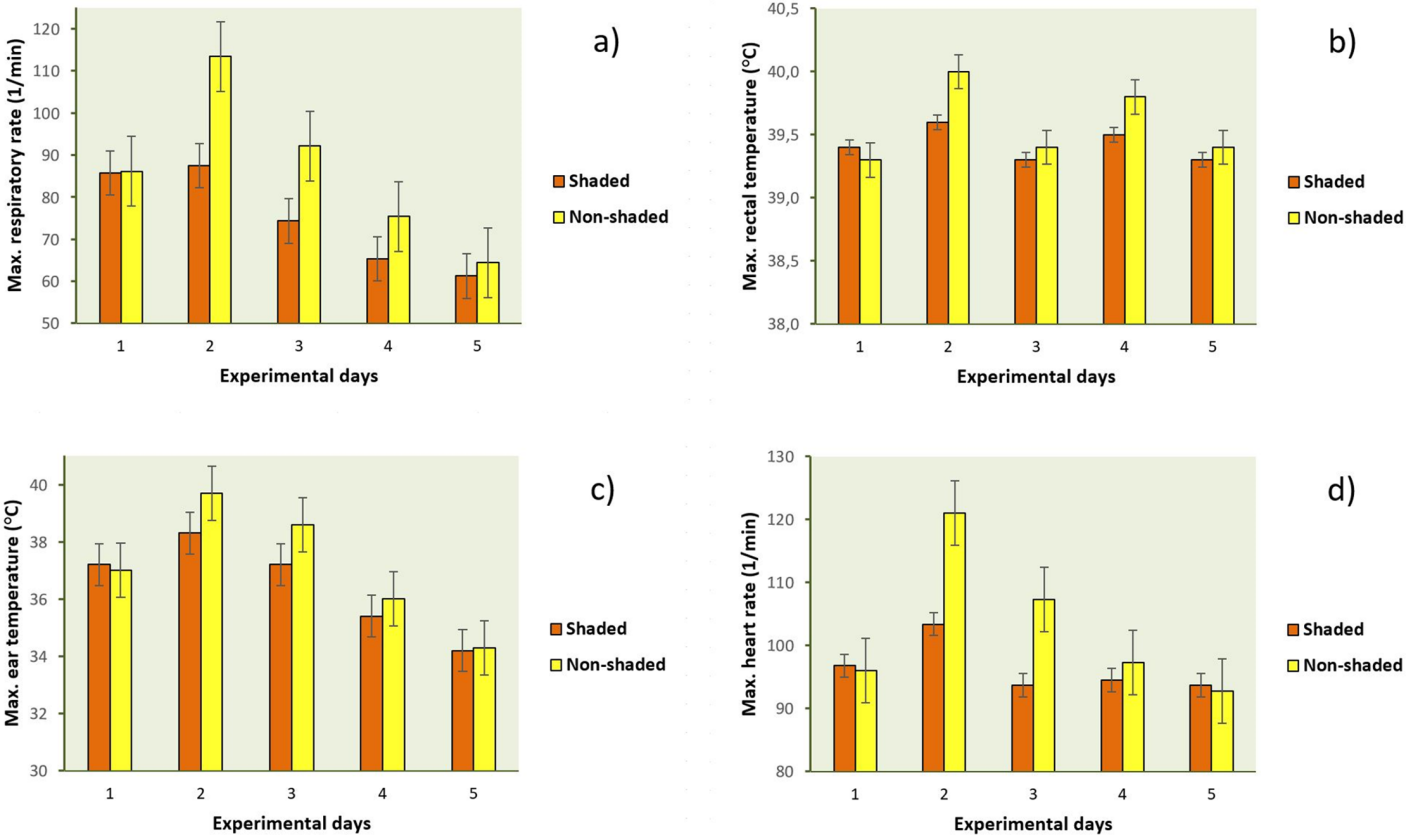
**Maximal values of respiratory rate (a), rectal temperature (b), ear temperature (c) and heart rate (d) of shaded and unshaded calves for the five experimental days.** Results are presented as means (±SD) based on individual data recorded with 30-min (for heart rate) and 4-h sampling frequency (for respiratory rate, rectal temperature and ear skin temperature). On day 1, calves for both groups were shaded. Day 2 = heat stress day.

**Fig 6:**
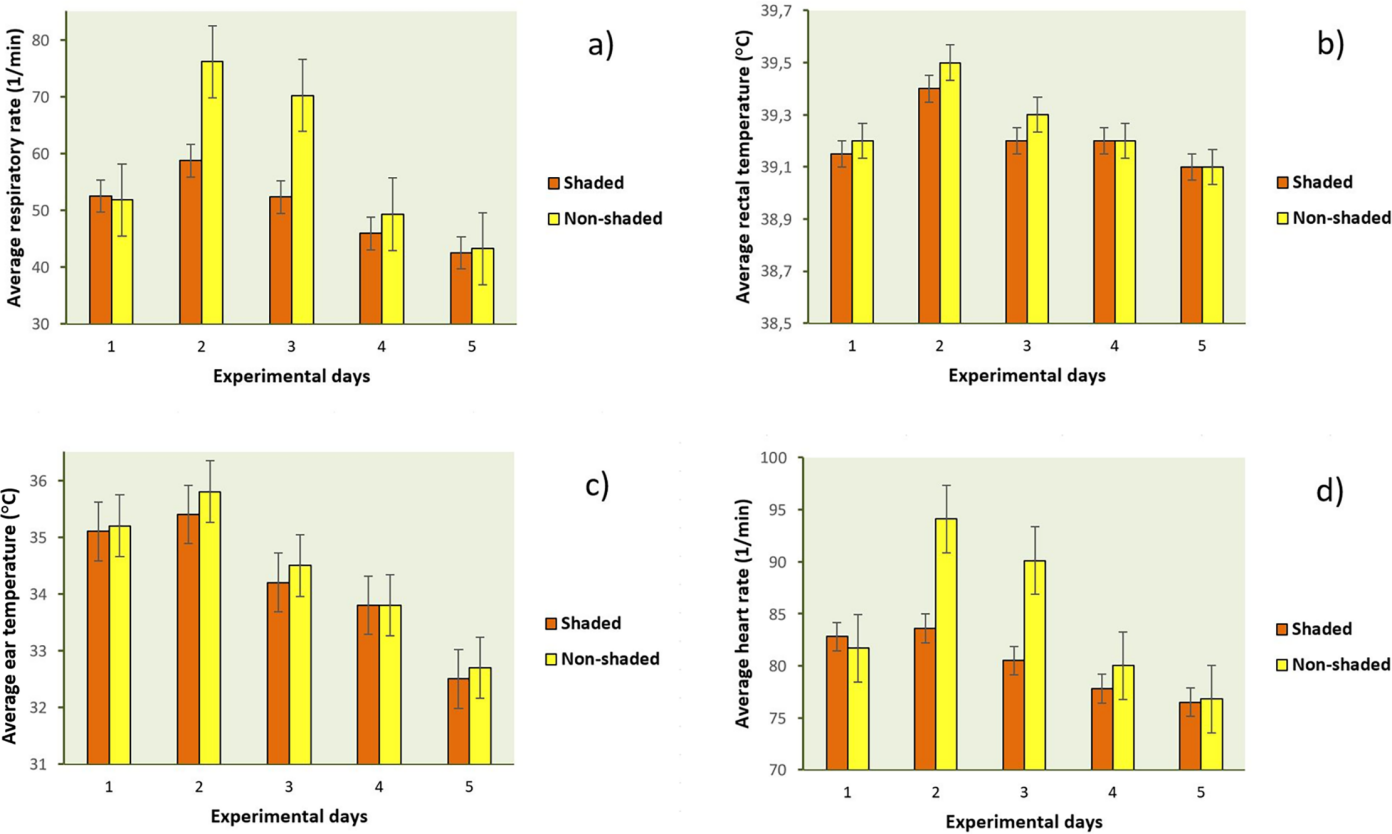
**Daily averages of respiratory rate (a), rectal temperature (b), ear temperature (c) and heart rate (d) of shaded and unshaded calves for the five experimental days.** Results are presented as means (±SD) based on individual data recorded with 30-min (for heart rate) and 4-h sampling frequency (for respiratory rate, rectal temperature and ear skin temperature). On day 1, calves for both groups were shaded. Day 2 = heat stress day.

Maximal and daily average values of physiological measures recorded for the 5-day experiment are presented in **Figs 5** and **6**. The analysis of animal-based parameters proved that supplemental shade should be provided for young calves to reduce acute, severe heat stress. Physiological parameters reflected high stress level of calves without shade on both days 2 and 3, and differences between groups were more pronounced in respiratory rate and heart rate compared to rectal or skin temperatures.

Our results are in accordance to previous findings on immediate physiological responses to short-term, severe heat load. In ruminants, acute heat stress has proven to induce rapid physiological responses within 24 to 48 h [17–19]. In the present study, during the heat stress day, maximal temperature and THI exceeded 43°C and 86 units in the unshaded hutch environment, respectively. An earlier study reported marked attempts to acclimate to acute heat stress at 37.7°C in 4-week old bull calves (which was the maximal temperature in the shaded hutch environment in our study) based on systemic indicators of stress, serum thyroxine and cortisol concentrations [20].

Lower respiratory rates in calves in shaded hutches are consistent with earlier observations that have shown in average 10.4 breaths/min lower respiratory rates (47.3 vs. 57.7 breaths/min) for shaded calves than for calves in hutches without shade when measured at 15:00 o’clock [14]. In the present study, differences were greater; by 25.9 and 17.8 breaths/min higher maximal respiratory rates in average on days 2 and 3, respectively (**Fig 5A**), in unshaded calves compared to shaded ones (*P* < 0.001 for both days). Daily averages of respiratory rates differed also between groups either on day 2 (*P* = 0.008) and day 3 (*P* = 0.010) (**Fig 6A**). Perhaps, calves of the present study were less adapted to extreme temperatures or to other features of their microenvironment. Another explanation for the substantial differences from previous results could be that the range of mean air temperature for individual sample days was 16.5 to 38.2°C in the cited paper, whereas animals were exposed to extremely high ambient temperatures during days 2 and 3 that ranged from 17.1 to 43.7°C for the unshaded hutch, and from 17.8 to 46.5°C for the unshaded pen environment in our study, respectively.

Although average rectal and ear temperatures did not reflect the positive effect of shading on day 2 (*P* = 0.865 and *P* = 0.760; **Fig 6B** and **6C**), significant, but not severe group differences were found for maximal body temperatures measured either in the rectum (0.5°C, *P* = 0.046) or on the ear skin (1.6°C, *P* = 0.035), respectively (**Fig 5B** and **5C**). Based on these findings sufficient daytime thermoregulatory ability of the experimental animals could be assumed (increased volume of water, and decreased feed intake) on the heat stress day. It was a limitation of our study that feed, and water intake were not measured. Previously, calf ear temperature reduced by about 0.3°C was found by positioning a shade cloth about 1.2 meters above the plastic calf hutches [14].

Following the heat stress day, differences in respiratory and heart rates were also meaningful with higher maximal and daily average values in unshaded animals for both parameters. Unshaded calves exhibited significantly higher maximal respiratory rates even on day 4 than calves with shading (**Fig 5A**, *P* = 0.025), suggesting their prolonged acclimatization to the challenging environment; however, based on this difference (10.1 breaths/min in average) a seriously impaired well-being of unshaded animals could not be assumed. Higher maximal and average heart rates of unshaded calves during day 2 (*P* < 0.001 and *P* = 0.010) and day 3 (*P* = 0.005 and *P* = 0.012) are possibly due to the increased physical activity associated with the high respiratory effort (**Fig 5D** and **Fig 6D**).

Although unshaded calves were exposed to significant daytime heat load during the afternoon hours on day 3 (maximal THI above 78, **Fig 4C**), even the maximal values of all animal-based parameters were in the physiological range. The available literature on critical cut-off points of different THIs of cattle focused only on adult cattle. Mader et al. [21], for example, determined 74 as a critical value of THI used in the present study in feedlot cattle based on panting scores, while others found THI 72 as critical in dairy cows [22]. In the light of previous findings, our results suggest that young calves would be expected to suffer less from heat stress than lactating cows that were also suffering from an additional metabolic heat output associated with milk production. Moreover, calves generate far less metabolic heat than cows and have a greater body surface area relative to internal body mass, which enables them to maintain thermal homeostasis more successfully than cows. However, cow studies usually assess production-related parameters of heat stress that cannot be measured in calves, which makes the comparison of cow and calf heat tolerance quite difficult.

It should be also noted, that despite extremely high daytime heat load observed for day 2 in this study, the drops of 18.2 and 16.4°C from daytime to nighttime temperatures on days 2 and 3, respectively, allowed calves to decrease core body temperature and thus the resumption of feed intake and normal physiological behavior. Although feeding and lying behavior were not investigated in this study, unshaded calves were usually in standing posture in the pen area at the feeding rack during the nighttime samplings, while shaded calves were mostly lying down in the hutches. It seems necessary to estimate thermal conditions which mean extreme stress for calves by determining upper critical values of meteorological indices. In these experiments, besides physiological measures, feed and water intake should be also recorded.

Our results emphasize that well-being of dairy calves kept in hutches exposed to direct sunlight can be impaired; nevertheless, the study might be limited due to the small number of calves used. It should be noted that the experimental environment and the arrangement of the calf hutches prevented the involvement of larger number of animals since bull calves were housed in three rows and each row had different orientation. To exclude orientation-related effects on thermal and physiological parameters calves from one row had to be selected. Age- and body weight-related differences between the study animals were also considered, thus calves from neighboring hutches were used (calves were placed into hutches based on the time of birth) that also limited the study population. Furthermore, we used only pre-weaned calves (up to 50 days of age) because weaning might have caused additional stress to the animals.

## Conclusions

Thermal variables correlated well with animal-based heat stress indicators and, in case of rectal temperature all correlations were stronger in the unshaded than in the shaded thermal environment. Ambient temperature correlated better with physiological measures than THI therefore should be used as measure of acute heat stress in continental regions. Shading of preweaned dairy calves may be adequate to alleviate heat stress in continental regions. Respiratory rate and heart rate support that the well-being of unshaded dairy calves can be impaired even during decreasing thermal load following a short-term heat stress event. However, to provide practical recommendations on shading of dairy calves, further studies might be necessary using larger number of animals.

## Supporting information

S1 Table Ambient temperature, relative humidity and THI values with 30-min sampling intervals during the 5-day study period in the shaded and non-shaded hutch environment. (XLSX)
