# Correction: Assessment of heat stress in 7-week old dairy calves with non-invasive physiological parameters in different thermal environments

**DOI:** 10.1371/journal.pone.0208528

**Published:** 2018-11-29

**Authors:** Levente Kovács, Fruzsina Luca Kézér, Ferenc Ruff, Viktor Jurkovich, Ottó Szenci

The following information is missing from the Funding section: This research was supported by the 17896-4/2018/FEKUTSTRAT grant of the Hungarian Ministry of Human Capacities.
